# Detección de errores de medicación mediante un programa de seguimiento y minimización en pacientes ambulatorios de Colombia, 2018-2019

**DOI:** 10.7705/biomedica.5544

**Published:** 2020-09-22

**Authors:** Manuel Enrique Machado-Duque, Jorge Enrique Machado-Alba, Andrés Gaviria-Mendoza, Luis Fernando Valladales-Restrepo, Ilsa Yadira Parrado-Fajardo, Mauren Ospina-Castellanos, Luisa Fernanda Rojas-Chavarro, John Alexander López-Rincón

**Affiliations:** 1 Grupo de Investigación en Farmacoepidemiología y Farmacovigilancia, Universidad Tecnológica de Pereira – Audifarma, S. A., Pereira, Colombia; 2 Grupo de Investigación en Biomedicina, Facultad de Medicina, Fundación Universitaria Autónoma de las Américas, Pereira, Colombia; 3 Atención Farmacéutica, Gerencia de Investigación Farmacoepidemiológica, Audifarma, S. A., Bogotá, Colombia

**Keywords:** errores de medicación, sistemas de registro de reacción adversa a medicamentos, farmacovigilancia, daño del paciente, Medication errors, adverse drug reaction reporting systems, pharmacovigilance, patient harm

## Abstract

**Introducción:**

El uso de medicamentos puede conllevar errores de medicación que desemboquen en la hospitalización del paciente, el aumento de los costos relacionados con la atención e, incluso, la muerte.

**Objetivos:**

Determinar la prevalencia de errores de medicación notificados en un sistema de información de farmacovigilancia en Colombia entre el 2018 y el 2019.

**Materiales y métodos:**

Se hizo un estudio observacional a partir del registro de errores de medicación de un sistema de farmacovigilancia que cubre a 8,5 millones de pacientes ambulatorios afiliados al sistema de salud de Colombia. Los errores se categorizaron en ocho grupos de la A (situaciones potenciales de error) hasta la I (error que pudo llevar a la muerte). Se hizo el análisis descriptivo y se estableció la prevalencia de los errores de medicación.

**Resultados:**

Durante los años 2018 y 2019, se reportaron 29.538 errores de medicación en pacientes ambulatorios, con una prevalencia general de 1,93 por cada 10.000 medicamentos dispensados. En el 0,02% (n=6) de los casos, se presentaron errores que llegaron a afectar a los pacientes y causaron daño (tipos E, F e I). La mayoría de los errores se asoció con la dispensación (n=20.636; 69,9%) y la posible causa más común fue la falta de concentración en el momento de dispensar los medicamentos (n=9.185; 31,1%). Los grupos farmacológicos más implicados en errores de medicación fueron los antidiabéticos (8,0%), los inhibidores del sistema renina-angiotensina (7,6%) y los analgésicos (6,0%).

**Conclusiones:**

Los errores de medicación son relativamente poco frecuentes y con mayor frecuencia se catalogan como circunstancias o eventos capaces de generar un error de tipo A. Muy pocas veces, pueden causarle daño al paciente, incluso, hasta la muerte.

## Abstract (en)

**Introduction:**

The use of drugs may involve medication errors leading to hospitalization, increased costs related to care, and even death.

**Objective:**

To determine the prevalence of medication errors reported in a pharmacovigilance information system in Colombia between 2018 and 2019.

**Materials and methods:**

We conducted an observational study based on the records of medication errors from a pharmacovigilance system covering 8.5 million outpatients affiliated with the Colombian health system. The errors were categorized from A (potential situations to error) to I (an error that could lead to death). We performed a descriptive analysis and established the prevalence of medication errors.

**Results:**

During 2018 and 2019, 29,538 medication errors in outpatients were reported with a general prevalence of 1.93 per 10,000 drugs dispensed. The errors that reached the patient and caused damage (types E, F, and I) occurred in 0.02% (n=6) of the patients. Most of them were related to the dispensation (n=20,636; 69.9%) and the possible most common cause was the lack of concentration at the time of dispensing (n=9185; 31.1%). The pharmacological groups most involved in medication errors were antidiabetics (8.0%), renin-angiotensin system inhibitors (7.6%), and analgesics (6.0%).

**Conclusions:**

Medication errors are relatively rare situations, generally classified as circumstances or events capable of generating the error (type A error). In low proportion, they can reach the patient and cause damage or even death.

Los medicamentos hacen parte de las intervenciones terapéuticas que buscan mejorar el estado de salud de los pacientes y su calidad de vida, así como promover la curación y reducir el sufrimiento, por lo cual su uso seguro es importante (1). Siempre debe tenerse en cuenta que se pueden producir errores de medicación, es decir, situaciones evitables que llevan al uso inapropiado del medicamento y pueden causar daño al paciente cuando está bajo el cuidado de un profesional de la salud. Estos errores son la principal causa prevenible de los efectos adversos, por lo que hoy se consideran un problema de salud pública (2). Los errores de medicación se clasifican asignándoles letras: la A se refiere a circunstancias o eventos que tienen la capacidad potencial de causar errores; la B, la C y la D, designan situaciones en que se presenta el error, pero este no causa daño; la E, la F, la G y la H, califican diferentes grados de daño, y la I, un error que pudo haber contribuido a la muerte del paciente o provocarla.

Desde hace cerca de 20 años, con la publicación del documento *To err is human: Building a safer health system* (3), se determinó que los errores de medicación podían implicar hospitalización, morbilidad, aumento de costos relacionados con la atención e, incluso, la muerte. Se evidenció que uno de los mayores problemas era la falta de comunicación asertiva entre el médico, el personal de salud, la farmacia y el paciente, lo que llevaba a dar información insuficiente sobre el medicamento correcto que debía administrarse, o sobre la dosis, la vía de administración o la frecuencia adecuadas. Los registros y las notificaciones de los errores de medicación son claves para detectar, reportar y construir estrategias que mitiguen el problema y mejoren la seguridad del paciente (1,4).

En algunos estudios se ha señalado que cerca del 7% de las prescripciones hospitalarias tienen errores de medicación (5), que el 5% de ellas se formula en la práctica ambulatoria, y que el 0,18% de los errores son graves (6). En un estudio llevado a cabo en pacientes hospitalizados en el primer nivel de atención en Colombia, se observó que casi todas las prescripciones tenían algún problema, como la falta de registro de la duración del tratamiento (97,3%), de la presentación y la forma farmacéutica (72,1%), o de la vía de administración (12,9%) (7). En otro estudio sobre dispensaciones ambulatorias en Colombia, se encontraron 14.873 errores de medicación, de los cuales 2.299 (15,5%) habían afectado al paciente (categorías C a I) y 79 (0,5%) habían ocasionado algún daño (categorías E a I) (8). Los reportes de errores en la medicación deben incrementarse, pues se estima que solo se registra el 5% de ellos (9).

Dadas las consecuencias de los errores de medicación en la práctica clínica, es imperativo adelantar nuevos estudios basados en la evidencia que sirvan de punto de partida para establecer su frecuencia y las implicaciones para los responsables de la atención de los pacientes. En este contexto, el presente estudio se propuso determinar la prevalencia de los errores de medicación notificados a un sistema de información de farmacovigilancia que cobija población ambulatoria afiliada al sistema de salud de Colombia.

## Materiales y métodos

Se hizo un estudio descriptivo, observacional, de carácter retrospectivo, a partir del registro de errores de medicación de Audifarma, S.A., operador logístico que dispensa medicamentos, aproximadamente, a 8,5 millones de pacientes ambulatorios afiliados a los regímenes contributivo y subsidiado, es decir, el 17,7% de la población de Colombia.

Se analizaron todos los errores de medicación reportados entre el 1° de enero de 2018 y el 31 de diciembre de 2019, sin otros criterios de inclusión o exclusión. Cada error es registrado por el funcionario que lo detecta (regente de farmacia, personal médico o de enfermería, o químico farmacéutico, etc.), en una aplicación virtual y es almacenado en una base de datos. Todos los errores son clasificados y revisados por un químico farmacéutico del programa de atención farmacéutica de Audifarma, S.A.

Los factores contemplados en la base de datos de errores de medicación del sistema de farmacovigilancia, son los siguientes cuatro:

1) ***Datos sociodemográficos:*** incluyen ciudad de dispensación, fecha y farmacia donde se reporta el error.

2) ***Error de medicación:*** se consigna el lapso de tiempo (en días) entre el evento y la fecha del reporte; el proceso involucrado (prescripción, dispensación, transcripción, administración, etc.); el momento de la detección; el consumo del medicamento por el paciente; la causa probable del error de medicación, entre otras, sobrecarga de trabajo, falta de concentración del funcionario, similitud fonética de los nombres de medicamentos o de las características del empaque, que faciliten el error (los llamados medicamentos “LASA”, *look alike, sound alike*).

3) ***Medicamento relacionado con el error de medicación:*** los diferentes fármacos fueron agrupados según el sistema de clasificación ATC (*Anatomical, Therapeutic Chemical*) creado por la Organización Mundial de la Salud.

4) ***Clasificación del error de medicación:*** se hizo según la taxonomía del *National Coordinating Council for Medication Error Reporting and Prevention* (NCCMERP) con las letras de la A a la I (cuadro 1) (10). Se considera que los errores clasificados de la E a la I, afectan al paciente y causan daño.

**Cuadro 1:** Frecuencia y prevalencia de los errores de medicación según su gravedad en el marco de un programa de farmacovigilancia que cubre pacientes ambulatorios de Colombia, 2018-2019

**Tipo**	**Gravedad**	**Total 2018-2019;** **n (%)**	**Prevalencia de EM por cada 10.000 ítems dispensados (2018-2019)**		**Frecuencia 2018; n (%) N=14.596**	**Prevalencia de EM/10.000 dispensaciones (2018)**	**Frecuencia 2018; n (%) - n=14596**	**Prevalencia de EM/ 10.000 dispensaciones (2018)**
A	Circunstancias o eventos que tienen la capacidad de causar errores	26.698 (90,4)	1,740		13.217 (90,6)	1,830	13481 (90,2)	1,669
B	Se produjo un error, pero el error no llegó a afectar al paciente.	1.519 (5,1)	0,099		771 (5,3)	0,107	748 (5,0)	0,092
C	Se produjo un error que afectó al paciente, pero no le causó daño.	1.263 (4,3)	0,083	582 (4,0)		0,080	681 (4,6)	0,084
D	Ocurrió un error que llegó a afectar al paciente y requirió seguimiento para confirmar que no resultara en daño para este o requiriera intervención para evitar daños.	52 (0,18)	0,003		23 (0,2)	0,003	29 (0,2)	0,003
E	Se produjo un error que pudo haber contribuido o causado un daño temporal al paciente y requirió intervención.	2 (0,01)	0,000		1 (0,0)	0,000	1 (0,0)	0,000
F	Se produjo un error que pudo haber contribuido o causado un daño temporal al paciente y que requirió hospitalización inicial o prolongada.	3 (0,01)	0,000		1 (0,0)	0,000	2 (0,0)	0,000
I	Se produjo un error que pudo haber contribuido o provocado la muerte del paciente.	1 (0,0)	0,000		1 (0,0)	0,000	0 (0,0)	0,000

Se elaboró un registro de los datos en Excel con toda la información y se analizó con el programa estadístico SPSS™, versión 25.0 (IBM, USA). Se hicieron análisis descriptivos estableciendo las frecuencias y las proporciones para las variables categóricas (cualitativas nominales), las medidas de tendencia central y de dispersión para las variables cuantitativas (continuas), la mediana y el rango intercuartílico (RIC) para las cuantitativas discretas. Se calculó la cantidad total de ítems (medicamentos) dispensados durante el periodo, con el fin de calcular la prevalencia de los errores de medicación según la siguiente fórmula: número total de errores / número de dispensaciones x 10.000 por año.

## Consideraciones éticas

El estudio se ajustó a las directrices para investigaciones sin riesgo contenidas en la Resolución 8430 de 1993 del Ministerio de Salud de Colombia, la cual establece las normas científicas, técnicas y administrativas para la investigación en salud; además, se respetaron los principios establecidos en la Declaración de Helsinki. Todos los datos se manejaron de manera anónima.

## Resultados

En los diferentes establecimientos farmacéuticos de Audifarma S.A. se dispensaron 152’809.646 ítems, 72’042.919 en el 2018 y 80’766.727 en el 2019.

Se reportaron 29.538 errores de medicación ambulatorios, 14.596 en el 2018 y 14.942 en el 2019, en los 417 establecimientos farmacéuticos (farmacias), ubicados principalmente en Bogotá (n=13.054; 44,2%), Cali (n=5.196; 17,6%), Medellín (n=1.293; 4,4%), Valledupar (n=1.103; 3,7%), Popayán (n=634; 2,1%), Tuluá (n=564; 1,9%), Palmira (n=541; 1,8%) y Santa Marta (n=493; 1,7%), y en otras 118 ciudades (n=6.660; 22,5%).

Se estableció una prevalencia de errores de medicación en pacientes ambulatorios de 1,93 por cada 10.000 medicamentos dispensados (2,02 por 10.000 en 2018 y 1,85 por 10.000 en 2019). Se reportaron 9.578 (32,4%) errores de medicación el mismo día en que ocurrieron, y los restantes se registraron, en promedio, 6,9 ± 13,5 días después.

La clasificación de los errores de medicación según la gravedad se presenta en el cuadro 1. Es notable la baja proporción de errores que llegaron a afectar al paciente y causaron daño; los errores más graves (de tipo E, F o I) solo se presentaron en seis (0,02%) pacientes y se relacionaron con captopril, lamotrigina, pamoato de pirantel, ácido valproico, albendazol o insulina.

**Figura 1:**
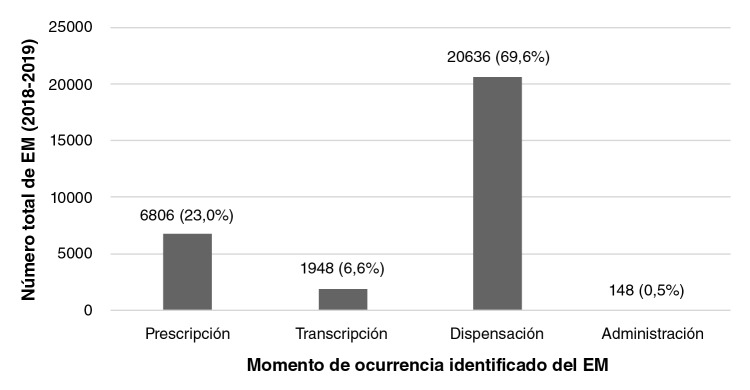
Momento de los errores de medicación detectados en un programa de farmacovigilancia que cubre pacientes ambulatorios en Colombia, 2018-2019

En cuanto al momento en que ocurrieron los errores, se pudo establecer que la mayoría de ellos se relacionaron con la dispensación (n=20.636; 69,9%) (figura 1). Al evaluar la posible causa del error, lo más común fue el reporte de falta de concentración en el momento de la dispensación (n=9.185; 31,1%), la similitud fonética del nombre de los medicamentos (n=7.025; 23,8%) o un almacenamiento incorrecto del producto (n=3.477; 11,8%).

Los medicamentos más frecuentemente implicados en los errores de medicación fueron losartán, levotiroxina, ácido acetilsalicílico y atorvastatina, en tanto que, al evaluar según la clasificación ATC, se encontró que los antidiabéticos, los inhibidores del sistema renina-angiotensina, los analgésicos y los antiulcerosos fueron los más comúnmente asociados con errores de medicación (cuadro 2).

**Cuadro 2:** Medicamentos y grupos de medicamentos más frecuentemente implicados en errores de medicación en el marco de un programa de farmacovigilancia que cubre pacientes ambulatorios en Colombia, 2018-2019

**Medicamento**	**Frecuencia total (2018-2019)**		**Frecuencia 2018**		**Frecuencia 2019**	
Principales 10 medicamentos relacionados con EM	**N=29.538**	**%**	**N=14.596**	**%**	**N=14.942**	**%**
Losartán	1.343	4,5	759	5,2	584	3,9
Levotiroxina	1.158	3,9	686	4,7	472	3,2
Ácido acetilsalicílico	1.027	3,5	548	3,8	479	3,2
Atorvastatina	1.017	3,4	606	4,2	411	2,8
Acetaminofén	929	3,1	395	2,7	534	3,6
Insulina	740	2,5	431	3,0	309	2,1
Esomeprazol	600	2,0	361	2,5	239	1,6
Antiácidos (hidróxido de aluminio)	551	1,9	285	2,0	266	1,8
Hidroclorotiazida	517	1,8	234	1,6	283	1,9
Naproxeno	513	1,7	232	1,6	281	1,9
Principales 10 grupos ATC relacionados con EM						
Antidiabéticos (A10)	2.376	8,0	1195	8,2	1181	7,9
Inhibidores del sistema renina-angiotensina (C09)	2.247	7,6	1283	8,8	964	6,5
Analgésicos (N02)	1.759	6,0	840	5,8	919	6,2
Antiácidos y antiulcerosos (A02)	1.684	5,7	920	6,3	764	5,1
Agentes modificadores de los lípidos (C10)	1.629	5,5	923	6,3	706	4,7
Oftalmológicos (S01)	1.354	4,6	552	3,8	802	5,4
Vitaminas (A11)	1.301	4,4	616	4,2	685	4,6
Tratamiento de tiroides (H03)	1.170	4,0	690	4,7	480	3,2
Antiinflamatorios y antirreumáticos (M01)	1.001	3,4	420	2,9	581	3,9
Broncodilatadores (R03)	980	3,3	446	3,1	534	3,6

## Discusión

Se pudo establecer la prevalencia en 1,93 errores de medicación por 10.000 medicamentos dispensados en uno de los registros de farmacovigilancia en las farmacias ambulatorias más grandes del país. El momento crítico fue la dispensación, pero la mayoría de casos se catalogaron como circunstancias o eventos con capacidad de causar el error. Los errores de medicación son un problema importante que merece la atención de las autoridades de salud, cuyas acciones deben enfocarse en garantizar la seguridad del paciente, además de lograr la efectividad deseada.

La prevalencia estimada de errores de medicación fue baja frente al número de ítems dispensados, y más del 90% se clasificó en la categoría A, lo cual, comparado con un reporte previo del mismo programa de farmacovigilancia, muestra avances significativos en la frecuencia del registro de errores por año. La proporción de errores creció de 1,02 a 1,93 por cada 10.000 medicamentos dispensados; los errores de tipo A representaban el 32,8% de los casos en el 2013. Lo anterior sugeriría la eficacia de la estrategia de minimización de errores de medicación, promoción del reconocimiento del error mismo y generación de una cultura de reporte adoptada por el operador logístico del programa de farmacovigilancia (8).

La proporción de errores de medicación ocurridos en el momento de la dispensación creció en comparación con el estudio del 2015 realizado por Machado-Alba, *et al.*, en una población similar, con 55,5% de los casos *Vs.* 69,9%, situación esperable en el ámbito de la atención ambulatoria, lo cual contrasta con los estudios hospitalarios en los que la mayoría sucede en el momento de la transcripción de la orden médica y en la administración (11). Lo relevante es que la frecuencia de errores en la administración se redujo casi a la mitad (0,9 *Vs.* 0,5% en el presente estudio); dichos errores suelen estar implicados en aquellas situaciones que pueden llegar a afectar al paciente y causar algún daño (8). La falta de concentración en el trabajo fue la causa más común reportada por el personal de la farmacia, lo cual contrasta con el estudio de Björkstén, *et al*., en un servicio hospitalario, quienes hallaron que la sobrecarga de trabajo, la comunicación poco clara de órdenes y la confusión de medicamentos debido al fenómeno LASA, fueron los principales factores relacionados con los errores (5,12).

Los medicamentos que crean confusión por sus nombres, su pronunciación o la similitud de los empaques (medicamentos LASA), pueden conducir a errores de prescripción, dispensación o administración. En algunos estudios se ha encontrado que este tipo de situaciones puede explicar entre el 7 y el 15% de los errores de medicación, valor que es inferior al reportado en este estudio (23,8%) (13,14). Este es uno de los aspectos más importantes que deben abordarse con los responsables de la asignación de los nombres de los medicamentos, con los químicos farmacéuticos responsables del etiquetado y el almacenamiento en la farmacia, con los laboratorios, de manera que fabriquen empaques distintivos para los medicamentos relacionados con el problema y, por último, con los dispensadores, para que observen todos los mecanismos de control diseñados para prevenir los errores de medicación.

Los medicamentos más frecuentemente implicados en errores de medicación fueron losartán, levotiroxina, ácido acetilsalicílico y atorvastatina, usualmente prescritos en la consulta ambulatoria para enfermedades crónicas no transmisibles y que tienen una alta rotación diaria en las farmacias, pero que, con excepción de la hormona tiroidea, afortunadamente no tienen un margen terapéutico estrecho y, en principio, tendrían menores riesgos para el paciente en caso de que lleguen a él (15).

En un estudio publicado por Nicolas, *et al.,* en Alemania se halló que los medicamentos más relacionados con errores de medicación fueron los antiinflamatorios no esteroideos, los beta bloqueadores y los antihipertensivos en general (16), situación similar a la de este estudio. Sin embargo, en comparación con el estudio realizado entre el 2005 y el 2013 en Colombia sí hubo un cambio, puesto que el acetaminofén y el metronidazol fueron los medicamentos más frecuentemente asociados con errores de todos los tipos, seguidos del losartán y la levotiroxina, que continúan estando entre los más comunes (8). También, se han presentado cambios entre los grupos ATC más frecuentes, puesto que se pasó de los del sistema cardiovascular a los del aparato digestivo y el metabolismo, especialmente los antidiabéticos, lo que podría estar asociado con el incremento del uso de insulinas en el país (17).

Las estrategias de mejoramiento de la seguridad en el uso de medicamentos en farmacias ambulatorias, requieren de la implementación de actividades de educación continua de todo el personal involucrado, en especial, de aquellos que están al final de la cadena de su manejo encargados de dispensarlos. Además, es necesario establecer protocolos de almacenamiento, registro, identificación especial de medicamentos LASA (incluso por parte de los fabricantes), dispensación y reconocimiento, reporte de los errores de medicación al Instituto Nacional de Vigilancia de Medicamentos y Alimentos (Invima) y búsqueda oportuna del paciente implicado para reducir el riesgo de que pueda causarle daño ((18,19) para que la mayoría se queden en errores de tipo A, como ha estado ocurriendo en los últimos años con el programa de farmacovigilancia aquí analizado. Sin embargo, sigue siendo necesario desarrollar estrategias de manera intensiva, en especial, para mejorar la oportunidad del reporte de los errores y reducir aquellos que puedan llegar a afectar al paciente, con el fin de fortalecer el sistema de farmacovigilancia establecido por el Invima (20).

El presente estudio presenta las limitaciones propias de los estudios observacionales: la información se obtuvo de un registro de reportes espontáneos de errores de medicación, el cual no tiene variables clínicas ni garantía de que los datos estén completos, pese a que son revisados y validados por los químicos farmacéuticos del programa. Además, solo se pudieron revisar los reportes realizados, que dependen de que el error sea detectado, pero quedaron excluidos todos aquellos que no fueron detectados por el personal del operador logístico, lo que constituye un potencial subregistro.

Con base en los resultados expuestos, se puede concluir que los errores de medicación fueron, por lo general, situaciones poco frecuentes (1,93 por cada 10.000 dispensaciones por año), que fueron categorizadas principalmente como circunstancias o eventos con capacidad de generar el error (tipo A), pero que solo pueden llegar a afectar al paciente y causar daños, e incluso la muerte, en una proporción muy pequeña. Además, están relacionados sobre todo con los medicamentos más utilizados en la práctica clínica, como antihipertensivos, antidiabéticos y antiagregantes plaquetarios. Estos errores se presentan especialmente en el momento de la dispensación, aunque también en el momento de la prescripción por parte del médico; la causa del error más frecuentemente reportada fue la falta de concentración de los funcionarios y las similitudes fonéticas de los nombres de los medicamentos.

Todo esto resalta la importancia de establecer y mantener sistemas de farmacovigilancia, de minimización de los errores de medicación, de capacitación y educación continua y de sensibilización de todas las personas que tienen que ver con las diversas fases de la cadena de manejo del medicamento, para evitarlos, reducir los riesgos, prevenir los daños y reportarlos de manera oportuna.
